# Urban ecosystem drives genetic diversity in feral honey bee

**DOI:** 10.1038/s41598-022-21413-y

**Published:** 2022-10-21

**Authors:** Aleksandra Patenković, Marija Tanasković, Pavle Erić, Katarina Erić, Milica Mihajlović, Ljubiša Stanisavljević, Slobodan Davidović

**Affiliations:** 1 Department of Genetics of Populations and Ecogenotoxicology, Institute for Biological Research “Siniša Stanković”-National Institute of the Republic of Serbia, University of Belgrade, Bulevar despota Stefana 142, 11060 Belgrade, Serbia; 2 Center for Forensic and Applied Molecular Genetics, Faculty of Biology, University of Belgrade, Studentski trg 16, 11000 Belgrade, Serbia; 3 Center for Bee Research, Faculty of Biology, University of Belgrade, Studentski trg 16, 11000 Belgrade, Serbia

**Keywords:** Ecological genetics, Urban ecology, Population genetics, Genetic variation

## Abstract

Urbanization can change biodiversity in both directions, positive and negative, and despite the rising global trend of urban beekeeping, little is known about the impact of urbanization on the genetic diversity of honey bees. We investigate how urbanization affects the genetic variability of feral and managed honey bee colonies that are spread throughout the entire city, even in highly urban areas, through genetic analysis of 82 worker bees. We found convincing evidence of high genetic differentiation between these two groups. Additionally, by comparing city samples with 241 samples from 46 apiaries in rural parts of the country, variations in mitochondrial *tRNAleu-cox2* intergenic region and microsatellite loci indicated that feral colonies have distinct patterns of genetic diversity. These results, with evidence that feral honey bees find niches within highly modified and human-dominated urban landscapes, lead us to conclude that urbanization is a driver of the genetic diversity of feral honey bees in the city.

## Introduction

Urban areas today are a frequent landscape feature and since 2010 more than half of the world’s population lives in cities^1^. The future of our planet is heading to urbanization as the world’s population increasingly moves from rural to urban centres, causing cities to grow with greater population density, even more in less developed regions^2^. Urban settlements are a highly complex and heterogeneous mosaic patchwork of habitat resources, where species face multiple challenges, from elevated pollution and noise, artificial and night illumination, and urban microclimate, to an accelerated reduction in habitat quality, including both abundance and diversity of many native species^3,4^. Cities often contain a high-density human population, a high number of exotic, invasive, or accidentally introduced species, few top predators, and inappropriate levels of nutrients and pollutants. Urbanization is related to global habitat degradation and loss, as evidenced by the increase in the amount of land occupied by urban expansion, and this trend is expected to accelerate in the coming decades. Land conversion and urbanization are perceived as major anthropogenic drivers of decline in the biodiversity of terrestrial species and act through a variety of mechanisms that decrease both reproduction and survival^5,6^.

The same trend is also evident in insects’ populations and is generally driven by land-use intensification and the conversion of natural habitats into agricultural or urban areas^7–9^. Special concern is given to pollinators such as bees, mainly due to their essential ecosystem services, their importance for modern agriculture, and the global pollination crisis where pollinating insect populations across multiple geographic locations and taxonomic groups are facing a significant decline in biomass and abundance^10–14^. Despite the generally negative factor of urbanization on pollinators, cities can support a surprising degree of bee biodiversity compared to other landscapes, reconsidering the traditional view of urban areas as “biological deserts”. The largest global analysis of the impact of land-use type and intensity on invertebrate pollinator biodiversity has shown significant pollinator species richness and changes in total abundance with both negative and positive effects^15^ further demonstrating that urban areas can support species-richness and abundant pollinator populations since some groups of bees might persist or even thrive in cities (e.g.^16–20^).

The presence of bee communities within cities does not imply that urban landscapes can compensate for lost natural habitats, they rather reveal that the heterogeneous landscape of urban systems creates a patchwork of potential habitats. Habitat resources in urban landscapes may not be equivalent to natural habitats regarding overall quality, patch size, connectivity, resource distribution, and resource phenology, but the presence of diverse and functional pollinator habitats cannot be denied. This variety of foraging and nesting sites can become a refuge for some bee species from increasingly less hospitable rural and suburban areas and the key to ecosystem function and resilience in cities under global climate change scenarios. For pollinators, agricultural intensification with high pesticide exposure, low floral diversity, and monocultures throughout the year may pose a greater risk than urbanisation itself^21^. A possible explanation for the higher diversity and abundance of bees in urban areas compared to rural areas is a greater number and variety of flowering plant species that provide more diverse nectar and pollen^19,22^. Additionally, urban species are often considered broad generalists, and honey bees fit this trend.

The history of human connection with western honey bee, *Apis mellifera*, globally the most prominent pollinator, dates back to at least nine thousand years^23^. Unlike other domestic animals, this species has not changed much because it was not as much “managed” but rather “kept” due to its biology^24^. Today, except in Africa, honey bees are maintained predominately in managed agricultural populations supported by beekeeping activity^25,26^. It was generally accepted that unmanaged (wild/feral) honey bee populations have disappeared in Europe since the 1980s after the introduction and spread of *Varroa* mites and the associated pathogens (e.g.^26^), but recent studies have shown that naturally nesting colonies of *A. mellifera* in its natural areal can still be found^27–31^. A honey bee colony is considered feral (FC) if it has returned to the wild, living naturally in an unmanaged state, and surviving without human intervention. However, it is very difficult to find and even estimate the number of colonies in the wild. Estimates based on predictions or derived from direct counting of wild/FCs can be unreliable due to several factors, making the assessment of their number, density, survival, and genetic background rather challenging^26,28^. Honey bees are cavity dwellers, and wild/FCs can find a range of suitable habitats. In nature, free-living colonies of *A. mellifera* are scattered, mostly in woodland areas, usually high in the trees, in inaccessible nesting sites. In urban areas, they can be found in natural and man-made cavities, such as trees, walls, and roofs, but also in most unlikely places such as shutter boxes and steel tubular electric poles^32^.

Even though wild/feral honey bee populations are often not considered and their existence is neglected in Europe, the presence of wild/FCs in European natural habitats is confirmed in several studies^27–31^. However, only one case of numerous unmanaged colonies is reported in urban areas in Europe^32^. The presence of wild/feral honey bee populations in Europe implores the need to increase knowledge about free-living *A. mellifera* populations^33,34^. For this study we used the existing database with reports of the numerous thriving honey bee colonies, living free and completely without human interference or treatment in Belgrade (more in^32^). The city of Belgrade, the capital and the largest city in Serbia and the second in Southeast Europe, has faced extreme overdevelopment and overbuilding in the past decade. Rapid development has induced unprecedented pressure on limited open and green space that must have had a negative effect on native species through fragmentation, isolation, and habitat destruction. In 2018, Belgrade had only 14.6% green spaces and only about 1.74% protected areas of the total city area^35^. Although the trend of careless urban growth, high pollution levels and environmental degradation is not declining, urban beekeeping in Belgrade is flourishing. In 2020 Belgrade had 80,000 beehives^36^. In the last 10 years, the number of hives and beekeepers in Belgrade has increased more than three times partially due to the financial support of both State and City Governments for beekeeping. Numerous workshops and practices for new urban beekeepers were organized within various beekeeping associations with the main goal of promoting urban beekeeping. The native honey bee *A. mellifera carnica* is productive and well adapted to specific local conditions, and Serbian legislation allows breeding of only this subspecies in order to preserve its autochthonous status^37^.

Urban areas are recognized as hotspots that drive changes in the environment and biodiversity on many levels. Urbanization is regarded as a new selective force, which imposes unique pressures that alter patterns of genetic variation in populations occupying urban habitats^38^. Although there is a rapidly growing interest in the effects of urbanization on genetic diversity, to our knowledge, studies that have focused on genetic differences between FCs and managed native honey bee colonies (MCs) in urban areas are almost non-existent. To expand the knowledge of the impact of urbanization on the genetic diversity of honey bees, we investigated feral and managed honey bees in the urban ecosystem and compared them with the data from rural areas. Urbanization poses challenges and opportunities for both MCs and FCs, but its evolutionary and ecological effects do not receive enough attention even though they are crucial due to their implications for conservation biology and understanding of ecosystem functions.

## Results

In this study, we analysed 40 samples (individuals) from 40 different urban FCs and 42 samples from seven urban apiaries (MCs). All samples with their origin (feral or managed), geographical coordinates, and determined mtDNA haplotypes are presented in Suppl. Table S1. The microsatellite data for each worker bee are presented in Suppl. Table S2.

### PCR–RFLP analysis

The PCR-amplified COI segment for RFLP analysis was 1029 bp long. Digestion with both the *NcoI* and *StyI* enzymes did not show characteristic recognition sites for the mtDNA lineage found in *A. m. macedonica* and it can be concluded that all individuals in our sample belong to *A. m. carnica.*

### Genetic diversity analysis of microsatellite loci

The standard parameters of genetic diversity for 14 microsatellite loci used in this study are presented in Table 1a. Our analysis showed that all the analysed parameters of genetic diversity are higher in FCs.

**Table 1:** Genetic diversity parameters: (a) microsatelites, (b) mtDNA *tRNA*^*leu*^*-cox2* intergenic region.

(a)											
Population	N	Agd	A	Am	Ar	Amr	H_O_	H_E_	MPD	RMP	G-W
BG feral	40	0.5771	8.071	6.57	1.67	11.86	0.4964	0.5771	8.0797	0.0125	0.66887
BG managed	41	0.5404	7.357	6.35	1.46	9.93	0.4930	0.5716	7.0253	0.0122	0.66484

(b)											
Population	N	H	Nps	Hd	π	RMP	MPD	Tajimas’s D			
BG feral	40	6	6	0.6256 ± 0.0467	0.001825 ± 0.001406	0.3900	0.974359 ± 0.675826	− 1.41098			
BG managed	42	5	6	0.5993 ± 0.0618	0.001966 ± 0.001480	0.4150	1.049942 ± 0.711495	− 0.77595			

(a) *N* number of individuals, *Agd* average gene diversity over loci, *A* average number of alleles, *Am* number of alleles based on a minimal sample size of 40 diploid individuals, *Ar* number of private alleles based on a sample of 40 diploid individuals, *Amr* mean allelic range, *H*_*O*_ observed heterozygosity, *H*_*E*_ expected heterozygosity, *MPD* mean number of pairwise differences, *RMP* random match probability, *G-W* Garza Williamson index.

(b) *N* number of individuals, *H* number of haplotypes, *Nps* number of polymorphic sites, *Hd* haplotype diversity, *π* nucleotide diversity, *RMP* random match probability, *MPD* mean number of pairwise differences.

All studied microsatellite loci were polymorphic, ranging from 2 alleles for the locus A28 to 15 alleles for the locus A107 (Suppl. Table S3). The average number of alleles per locus was 8.07 in FCs and 7.36 in MCs (Table 1 and Suppl. Table S3). The average genetic diversity, measured as expected heterozygosity (He), was almost the same in both analysed groups, while both groups showed a lower value for observed heterozygosity (Ho) (Table 1).

Most of the analysed loci did not exhibit deviation from the HW equilibrium in both groups. After Bonferroni correction, at the FCs loci A7, A28, and A43, a statistically significant deviation from HW equilibrium was retained compared to one locus (A43) in MCs (Suppl. Table S3). For the expected homozygosity parameter (θ_H_) half of the loci in one of the types of honey bee colonies analysed (feral vs managed) exhibited higher values compared to the other, while the θ from mean homozygosity was almost equal in both FCs and MCs from Belgrade (Suppl. Table S4a). The *F*_*IS*_ parameter demonstrates that the Belgrade FCs exhibits higher values of inbreeding compared to the MCs (Suppl. Table S4b). The inbreeding coefficient (*F*) analysis showed that the F values are highly dependent on the estimator used for the analysis (Suppl. Table S5). According to the estimators with the highest correlation (TrioML/DyadML), FCs exhibits higher *F* values compared to MCs. In addition, the TrioML estimator exhibits the lowest variance for the FCs and the DyadML estimator shows the second-lowest variance for the same parameter. Statistically significant linkage disequilibrium was observed within 13 loci pairs in FCs and 10 loci pairs in MCs. Disequilibrium of the linkage for the pair A79/A7 was detected in both groups and in the MCs for the pair Ap249/B124 which are recognized as selectively adaptive loci^39^ (Suppl. Table S6).

### Genetic diversity of mitochondrial *tRNA*^*leu*^*-cox2* intergenic region sequences

**Figure 1:**
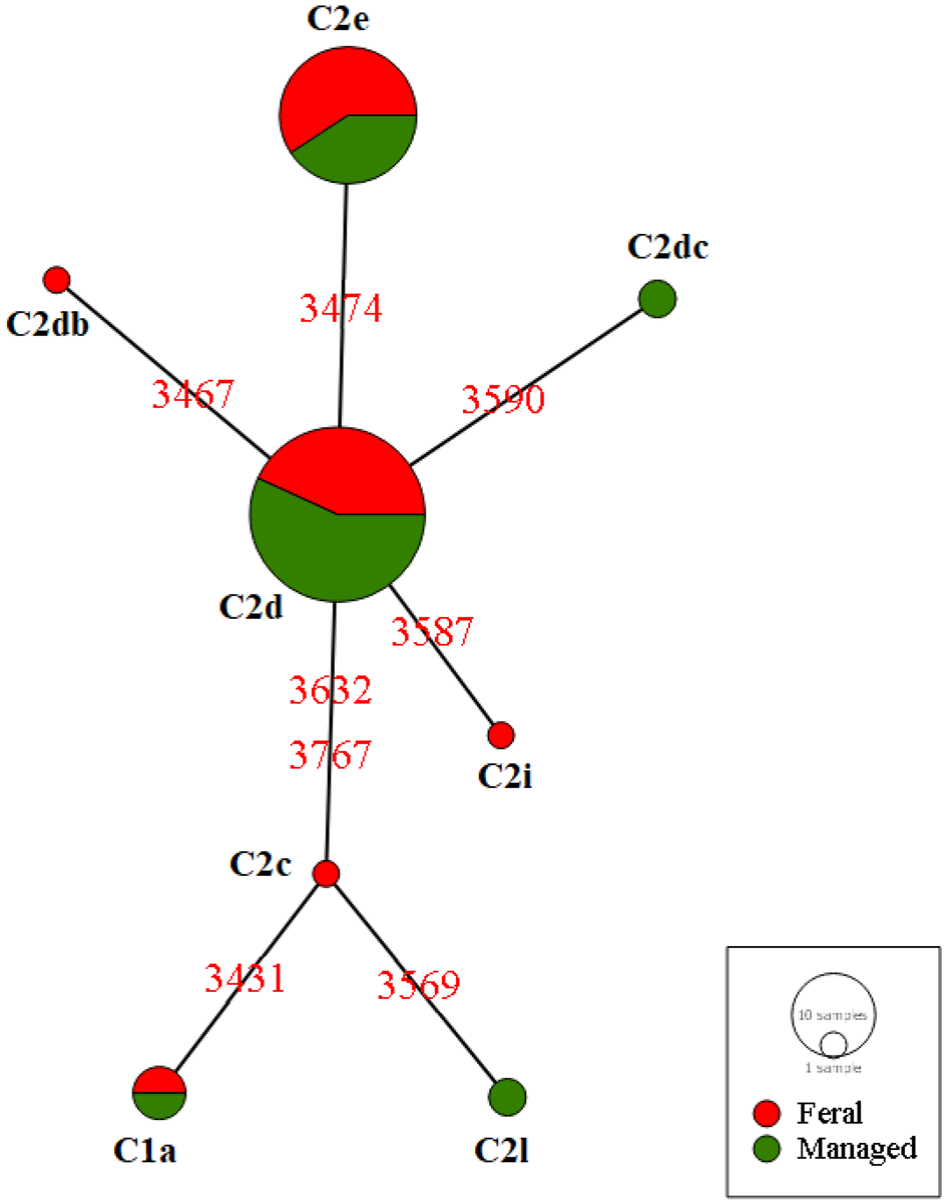
Median-joining phylogeographic network of mtDNA haplotypes detected in two types of honey bee colonies (feral and managed) in the territory of Belgrade based on the variability of the *tRNA*^*leu*^*-cox2* intergenic sequence. The size of the node is proportional to the number of individuals. The numbers represent variable nucleotide positions between different haplotypes.

Analysis of intergenic sequence variability of *tRNA*^*leu*^*-cox2* revealed that a total of eight different haplotypes belonging to the eastern Mediterranean C lineage were present in Belgrade (Fig. 1 and Suppl. Table S1). Seven of the eight detected haplotypes have been previously described, while the haplotype defined by the transition at the position 3590 is novel and hereafter named C2dc according to the current nomenclature. This haplotype was detected only in the MCs. Interestingly, the haplotype C2db (first described by Tanasković et al.^40^, from North Serbia), was detected only in FCs. Sequences generated in this paper are deposited in GenBank, accession numbers ON187787-ON187868.

Although both groups share a similar number of haplotypes, three were found only in FCs and two exclusively in MCs, while the most frequent haplotypes were found in both groups (Table 1, Suppl. Table S1, and Fig. 1). FCs exhibit somewhat higher values for the haplotype diversity parameter, while MCs exhibit higher values for the π and MPD parameters (Table 1). Higher π and MPD values suggest that the detected haplotypes in MCs are genealogically more distant than those in FCs, as can be seen in Fig. 1 and are also caused by the absence of the C2c haplotype in MCs. Interestingly, the RMP parameter is lower in FCs, further suggesting a higher diversity that can be found within this group.

### Genetic differentiation inferred from the microsatellite loci and mtDNA analysis

**Figure 2:**
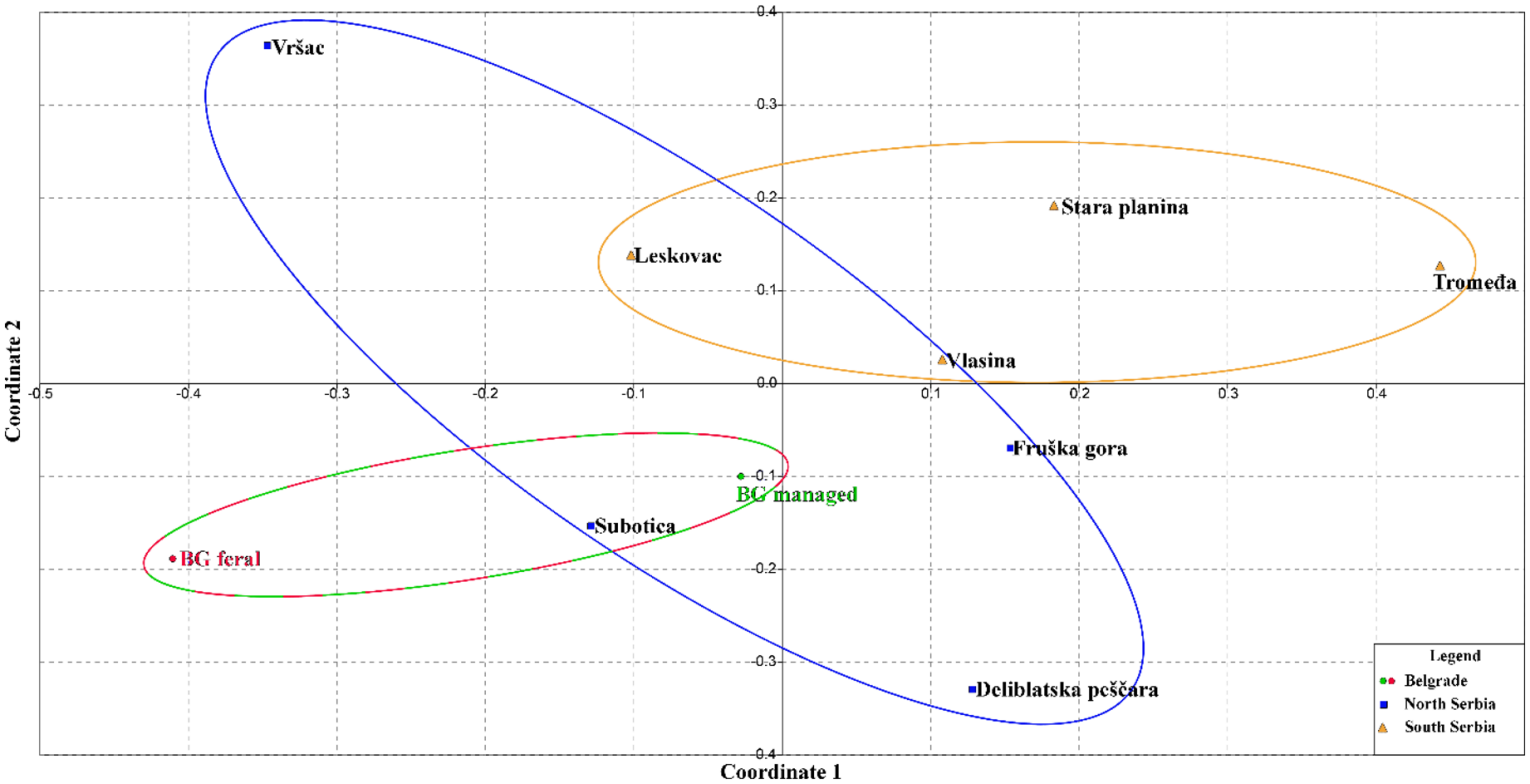
Non-metric multidimensional scaling plot of *F*_*ST*_ distances between two types of honey bee colonies from Belgrade and honey bee colonies originating from different regions of Serbia based on the variability of 14 microsatellite loci. The goodness of fit is expressed with the stress value, which is 0.1909 for this data set. Population pairwise *F*_*ST*_ values are presented in Suppl. Table S7.

Genetic differentiation based on 14 microsatellite loci between two groups of Belgrade honey bee colonies with other MCs from Serbia, was evaluated by comparing the pairwise *F*_*ST*_ distances, and the results are presented in Suppl. Table S7a. Pairwise *F*_*ST*_ values between the Belgrade FCs and all MCs colonies exhibit the highest and statistically significant pairwise differences, including a statistically significant pairwise difference with Belgrade MCs (p = 0.000; Suppl. Table S7a, Fig. 2 and Suppl. Fig. S1). When different localities from the north and south parts of Serbia are grouped, Belgrade FCs occupy a distant position compared to all MCs analysed (Suppl. Fig. S2) and exhibit the highest and statistically significant values for pairwise *F*_*ST*_ (Suppl. Table S7b).

**Figure 3:**
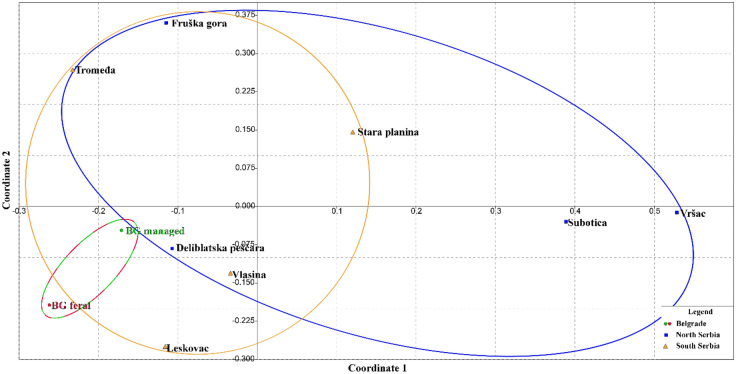
Non-metric multidimensional scaling plot of *F*_*ST*_ distances between two types of honey bee colonies from Belgrade and honey bee colonies originating from different regions of Serbia based on the variability of *tRNA*^*leu*^*-cox2* intergenic sequence. The goodness of fit is expressed with the stress value, which is 0.1120 for this data set. Population pairwise *F*_*ST*_ values are presented in Suppl. Table S8.

Genetic differentiation at the mtDNA level based on the analysis of pairwise differences in *F*_*ST*_ revealed its absence of genetic differentiation between the Belgrade FCs and MCs (Suppl. Table S8a). However, the same analysis showed the existence of statistically significant *F*_*ST*_ values between the FCs and half of the analysed MCs (SP, FG, S and Vr), while the statistically significant *F*_*ST*_ values were observed between Belgrade MCs and two managed colonies (S and Vr). Other tested pairwise *F*_*ST*_ values were low and statistically unsupported (Suppl. Table S8a, Suppl. Fig. S3 and Fig. 3). Grouping of Serbian localities into North and South and comparison with FCs and MCs from Belgrade did not reveal any significant differences based on pairwise *F*_*ST*_ values although the colonies analysed tend to occupy different positions on the MDS plot (Suppl. Table S8b and Suppl. Fig. S4).

AMOVA analysis of mtDNA *tRNA*^*leu*^*-cox2* intergenic region sequence variability revealed the lack of variation between two types of Belgrade honey bee colonies (Table 2). When other MCs from the north and south parts of Serbia were included, we found variation among the sampled localities, but that the majority of the variation (94.35%) remained within them (Suppl. Table S9). AMOVA also showed that when Belgrade honey bee colonies are grouped as one group and compared with north or south parts of Serbia, the percentage of variation between groups is higher than if they are observed as separate groups (0.27% vs − 1.65%, Suppl. Table S9) suggesting that Belgrade honey bees can be viewed as one population considering the pool of mtDNA. On the other hand, the AMOVA analysis based on the variability of the microsatellite loci revealed greater differences between Belgrade FCs and MCs (Table 2).

**Table 2:** Outcomes of the analysis of molecular variance (AMOVA) based on the variability of (a) mtDNA *tRNA*^*leu*^*-cox2* intergenic region and (b) 14 microsatellite loci analysed in two types of honey bee colonies.

Source of variation	df	Sum of squares	Variance components	Percentage of variation
**(a)**				
Among populations	1	0.403	− 0.00253	− 0.50 (*p* = 0.47297)
Within populations	80	40.524	0.50655	100.50
Total	81	40.927	0.50402	
**(b)**				
Among populations	1	9.032	0.05598	1.39 (p = 0.02188)
Among individuals within populations	79	355.37	0.53004	13.17 (p = 0.00000)
Within individuals	81	278.5	3.43827	85.44 (p = 0.00000)
Total	161	642.901	4.02429	

Considering microsatellites, the variation between FCs and MCs is observable and statistically significant (Table 2). When colonies from other regions of Serbia were included in AMOVA, the variation among analysed localities remained similar, while the variation among individuals within populations became lower (13.17% vs 6.64%) and within individuals became higher (85.44% vs 91.91%) (Table 2 and Suppl. Table S9). Interestingly, when FCs and MCs from Belgrade were observed as a group and compared against colonies from either north or south parts of Serbia, the percentage of variation among groups was lower than when these two types of Belgrade colonies were considered as separate groups (0.26% vs. 0.32%), suggesting that on the level of microsatellites, FCs and MCs can be regarded as separate groups (Suppl. Table S9).

### Population structure based on microsatellite loci in Belgrade sample set

**Figure 4:**
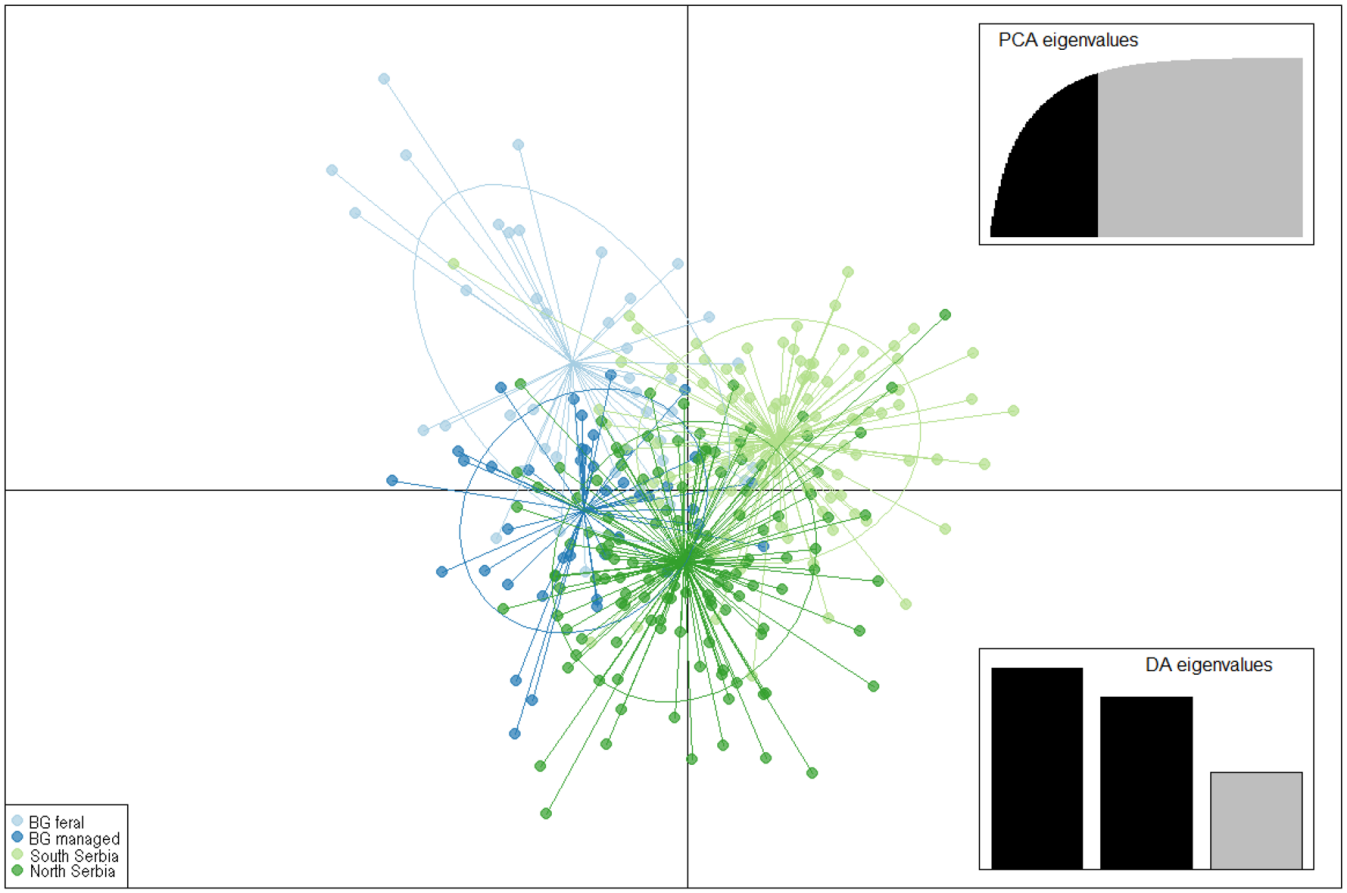
Discriminant analysis of principal components in which LDA was performed on the first 62 PCs (out of 197 PCs) cumulatively conserved 98.9% of the total variance. The first and second linear discriminants are presented in the plot.

DAPC analysis showed that individual honey bees from different colonies and different regions of Serbia position in overlapping clusters, while feral honey bees from Belgrade occupy a distinct position on the plot defined by the first two DA eigenvalues (Fig. 4). In addition, Belgrade FCs tends to take a more isolated position compared to MCs (Fig. 4). When viewed in three dimensions, DAPC reveals a more pronounced differentiation between FCs and MCs from Belgrade and the geographical stratification of samples from north and south could also be observed (Suppl. Fig. S5).

**Figure 5:**
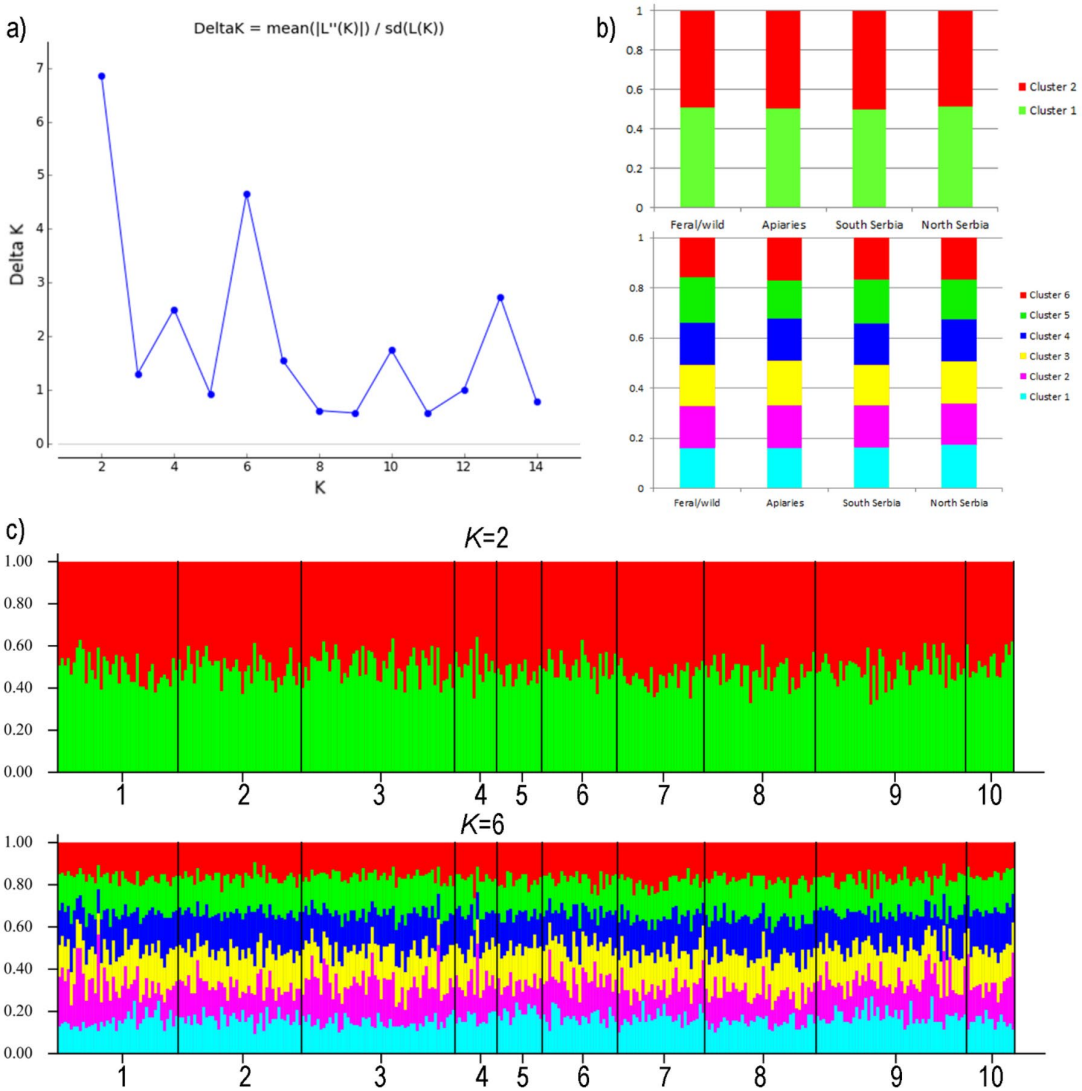
(**a**) Δ K mean for the assumed number of genetic clusters. (**b**) Proportions of inferred STRUCTURE clusters (K = 2 and K = 6). (**c**) Proportions of the inferred STRUCTURE clusters (K = 2 and K = 6) from the individuals. 1—BG feral, 2—BG managed, 3–6—South Serbia (3—Leskovac, 4—Vlasina, 5—Tromeđa, 6—Stara planina), 7–10—North Serbia (7—Fruška gora, 8—Subotica, 9—Deliblatska peščara, 10—Vršac).

DAPC analysis suggested the existence of 15 genetic clusters (Suppl. Fig. S6). In both cases, FCs and MCs from Belgrade share most of the ancestry and some of the genetic clusters were found in only one type of colony. A different approach using STRUCTURE estimated the existence of two genetic clusters as the most likely scenario (*K* = 2, Fig. 5 and Suppl. Fig. S7). Evanno's method suggested that *K* = 6 also exhibits high probability, but, nevertheless, the distribution of ancestral genetic clusters is equal in all assessed regions and in both types of colonies (Fig. 5). The observed equal distribution of ancestral genetic clusters suggests that all Belgrade honey bees and those from other parts of Serbian share the same ancestry.

### Relatedness

Relatedness was estimated for 82 worker bees representing each honey bee colony on the territory of Belgrade. For the evaluation of relatedness, the seven estimators available in the COANCESTRY^41^ program were used (Suppl. Table S10). Although the LynchRd estimator exhibited the lowest variance (Suppl. Table S11), the TrioML estimator^42^ was used to represent the relatedness among the pairs of worker bees (dyads), i.e. colonies. TrioML exhibited the second-lowest variance and together with the DyadML estimator (an estimator with the fourth-lowest variance) showed the highest correlation, further supporting its usage as the estimator of choice (Suppl. Table S11). In Table 3a the mean value of the relatedness *r*_*xy*_ among analyzed colonies based on 95 CI for the TrioML estimator is presented. According to 95 CI for TrioML, there are no colonies with a full sibling or parent/offspring relationship and on average each colony has 0.28 related colonies of second-order and almost one related colony of third-order (Table 3a and Suppl. Table S12). On average, each colony is related to 1.26 other colonies, and each colony from Belgrade has 0.67 related colonies from FC types and 0.59 related colonies from MCs (Table 3 and Suppl. Table S12). In the FCs sample, 35% of the colonies analyzed have no related colonies, while 39% of the MCs have no related colonies (Suppl. Table S12). Although on average FCs and MCs exhibit a similar number of related colonies, FCs have more related colonies within their group than they have related colonies within the MCs (Table 3b). These results indicate that the FCs are more related to other FCs than they are to the MCs.

**Table 3:** Relatedness estimates for individual pairs of diploid individuals and average numbers of colonies that have full-sib, second-order, and third-order relationships for each colony in the sample and for both wild/feral and managed colonies according to the TrioML estimator.

(a) Relatedness estimates for honey bees colonies in Belgrade								
	*r*_*xy*_	Variance	Afs	Asor	Ator	Ar	AFr	AMr
Relatedness (CI95)	0.422	0.0328	0 ± 0	0.28 ± 0.60	0.98 ± 1.32	1.26 ± 1.66	0.67 ± 0.97	0.59 ± 0.98

(b) Relatedness among two different types of colonies								
	N	Nnr	Nr	Ar	Afr	Amr		
Feral	40	14	35.00	1.35	0.75	0.60		
Managed	41	16	39.02	1.17	0.59	0.59		

(a) *r*_*xy*_—mean relatedness for individual pairs of diploid individuals; Afs—average number of full siblings per individual; Asor—average number of second-order relatives per individual; Ator—average number of third-order relatives per individual; Ar—average number of relatives (full-sib/parent–offspring relationships, second-order relationships and third-order relationships), AFr—average number of relatives in the sample of wild/feral colonies, AMr—average number of relatives in the sample of managed colonies.

(b) N—number of colonies; Nnr—Number of colonies without related colonies; Nr—percentage of colonies that are not related with any other colonies; Ar—average number of related colonies per colony; Afr—average number of related feral colonies per colony; Amr—average number of related managed colonies per colony.

## Discussion

This study represents the first population genetics study on honey bee colonies in highly urban landscapes based on their managed status. Our results provide important evidence to the scientists and beekeepers interested in free-living wild/feral colonies that survive and thrive despite the degradation and loss of natural forest habitat, numerous pests and pathogens^34,43^. Overall, our findings show that urban areas can serve as an important habitat suitable for free-living unmanaged honey bees and that feral urban honey bees exhibit observable genetic differentiation compared to MCs from the same territory. To describe genetic diversity and quantify in both within and between colonies, genetic variation, 14 microsatellite loci, and the mitochondrial highly variable intergenic region between the *tRNA*^*leu*^ and cytochrome c oxidase subunit II (*tRNA*^*leu*^*-cox2*), were studied. These molecular markers are widely used in population genetics, taxonomy, systematics, and biology of honey bee conservation, and, in our study, they clearly show that two species of bee colonies from the same territory differ from a molecular point of view.

Both types of sampled colonies of *A. m. carnica* in urban ecosystems possess relatively high levels of genetic variability, but values for both microsatellite and mtDNA genetic markers are slightly higher in urban FCs. Genetic diversity defined as any measure that quantifies the magnitude of genetic variability within a population is a crucial source of biodiversity and as such recognized as one of three major components of biodiversity worthy of conservation under IUCN^44^. It provides raw material for evolution that through natural selection drives the process of speciation and contributes to the tight relationship between heterozygosity and population fitness. Genetic diversity is essential for the long-term survival of populations^45–47^. The presence of broad genetic variation is crucial in overcoming challenges, particularly in a rapidly changing environment threatened by growing human impacts and accompanying habitat loss and climate change. In honey bees, there is a strong body of evidence that within-colony genetic diversity reduces the negative impacts of pathogens and parasites^48–53^, increases pollen foraging efficiency^54^, and improves the control of hive temperature^55,56^. The reduction in genetic diversity within colony may also affect the capacity of honey bee populations to adapt to new threats, which is in line with the global decline of managed colonies. Microsatellite analysis showed that, compared to all other types of colonies analysed in Serbia, FCs in most cases have higher values for different parameters of genetic diversity. Results based on the analysis of 14 microsatellite loci show that when FCs are compared with MCs from Belgrade and the Serbian countryside^57^, in most cases they possess higher values of genetic diversity parameters. These results are not entirely unexpected—from the last decades of the twentieth century, commercial beekeeping has dominated in Serbia and has completely suppressed more sustainable traditional ways. Modern beekeeping practices, such as commercial breeding, the introduction of non-native queens and colony trade, selection and migratory beekeeping, rapidly change the genetic diversity of managed honey bees in Serbia^40,57–59^. A similar impact of migratory beekeeping and honey bee trade on the genetic diversity of managed honey bees has been observed in Turkey with the admixture of different subspecies and local honey bee populations of Anatolia and Thrace^60^. The AMOVA analysis based on microsatellite loci revealed a high level of variation both within individual bees from colonies and within individual bees from a group of colonies from the same populations/locality. This reveals a population structure with genetically distinct individuals and more sexual outcrossing than we would expect. The results indicate that the local colonies maintain specific allelic variants, and a small amount of variation shown among groups (1.39%) indicates a genetic similarity, homogeneity between the populations/localities, and gene flow between them. Serbian honey bee breeders occasionally import *A. m. carnica* queens, usually from Slovenia, and widely exchange them and their selected queens with beekeepers from all parts of Serbia. The queen trade is very intensive in this region, and introductions of queens of different origins, not locally adapted, could be the source of the less relatedness detected for MCs (Table 2). Additionally, gene flow derived from extensive beekeeping mobility may also contribute to changes in the genetic pool of managed honey bee populations and drive the loss of their genetic diversity, since particularly rare alleles are lost in a reduced gene pool^57,60–62^.

**Figure 6:**
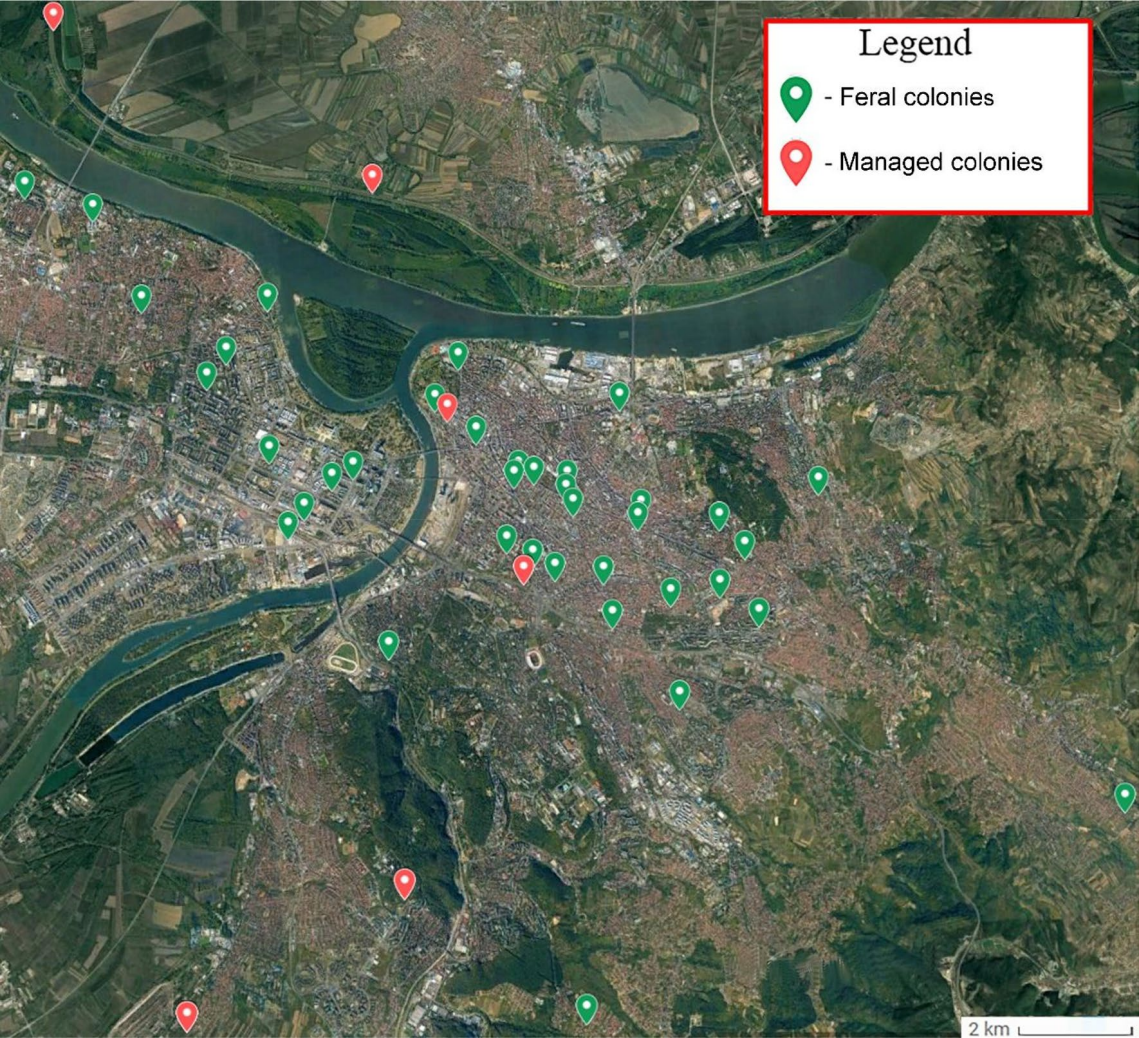
Sampling locations for feral and managed honey bee colonies in Belgrade. The base satellite imagery was obtained from Google Earth, and Adobe Photoshop CC 2015 (https://www.adobe.com/products/photoshop.html) was used to map the data by S. D. The locations overlying the map were obtained from the field using a GPS device (model no: Garmin eTrex 22x).

The results point out that urban feral and managed colonies have different patterns of departures from HW equilibrium and LD calculated for microsatellites loci, which may be an indication of various selection pressures and genetic differentiation of the colonies. When these two types of colonies were compared on microsatellite levels, it was shown that FCs possessed on average a greater number of alleles, while MCs possessed greater variability of alleles for the loci Ap249 and B124 (Suppl. Table S3). These two microsatellite loci, among all investigated loci, are considered to be selectively adaptive. The loci Ap249 and B124 are associated with genes involved in stress and immune responses, and could potentially reflect selective processes^39^. The Ap249 locus is linked to the protein-coding gene *Aos1* related to a protein modification involved in various cellular processes that include stress, the immune response to infection, and plasticity of the nervous system, while the B124 locus is associated with the protein-coding gene *dpr7* that encodes immunoglobulin proteins. Although MC types have 10 pairs of loci in LD in contrast to 13 pairs of loci in FCs, the managed group has LD for the Ap249/B124 loci. The selection pressure on the adaptive alleles Ap249 and B124 of the respective microsatellite loci can be the result of artificial selection for the resistance/tolerance traits, or natural selection possibly associated with genes involved in local adaptation to pathogens and parasites. Unmanaged honey bees often show specific adaptations to the local surroundings, including various parasites and diseases as well as higher pathogen pressures in natural habitats, and these selective pressures can be a reason for the smaller number of alleles detected for the adaptive loci Ap249 and B124. The allelic variants present in the FC population have already been subject to natural selection due to the conditions in which the FCs live. Most MCs have a low probability of survival without chemical treatments against the mite *Varroa destructor* (and other parasites) and depend on pesticides and antibiotics treatment multiple times per year. These control treatments limit the selective pressure, preventing coevolutionary processes towards a stable host-parasite relationship^63^. Additionally, modern beekeeping practices rely on the selective breeding of queens with desirable traits, while resistance to pathogens and parasites is recognized as a highly valuable trait^64^. Owing to treatments against parasites and mites, the selection pressure in MCs is not as strong as in FCs and it could be a reason for the greater variability of these adaptive alleles in FCs. Strong selection by breeders and beekeepers for these favourable traits may be one of the reasons for the existence of linkage disequilibrium at these loci. Simultaneously, managed honey bees are protected from other selective pressures as beekeepers provide supplemental food, shelter, and therapies against pathogens and parasites. Feral bees, however, experience various selective pressures relative to managed honey bees and thus may provide insight into the processes of adaptation to local conditions, in this case, to an urban area, which highlights the need for long-term honey bee studies across a variety of urban settings. Although both managed and feral honey bees experience different sets of selective pressures, honey bees from Serbia have the same genetic history and share the same ancestry (Fig. 6). The same observation of shared ancestry can be deduced based on mtDNA variability. Our results indicate that both FCs and MCs, although characterized by the presence of rare haplotypes, are not genetically well-differentiated since the analysis of the *tRNA*^*leu*^*-cox2* intergenic region did not show high enough resolution for this matter. Analysis of complete mitogenomes from these two types of honey bee colonies could potentially reveal greater differences, especially considering the fact that coding region of the mtDNA are a subject of natural selection that can favour some of the mtDNA lineages more than others.

Interestingly, our data showed that when feral honey bees are left unattended and without human intervention, they naturally tend to have more related colonies among themselves than among MCs. The results indicate that the FCs in Belgrade can be considered a self-sustaining population with a low gene flow from MCs. Our results suggest that swarming from the MCs is not the only source of FCs detected on the territory of Belgrade. However, it remains unclear whether these colonies represent swarms that escaped from managed apiaries, able to adapt to the urban environment after a long period of life free of human intervention, or they are indeed wild populations. Unfortunately, since this is the first study of the genetic structure of the free-living honey bee population in this highly urban area, making a comparison between the previous and the current status is impossible. The data presented here are a stepping stone in the continuous monitoring of the genetic variability of feral honey bees, which is the future goal of our research. Finally, in this study we investigated the effects of urbanization on the genetic diversity and structure of honey bees, and based on highly variable genetic markers, genetic differentiation was found between FCs and MCs. However, little is still known about the connections between genetic variation among honey bees and anthropogenic influences on the environment. Given extensive knowledge on how environmental disturbance negatively affects the presence and abundance of native bees in urban habitats, it would be expected that a high level of urbanization will accelerate the loss of genetic diversity in urban FCs, but our results show otherwise. The effects of urbanization on insect pollinators may also be expected to differ among different urban areas. Every urban area has a unique geography, climate, policy, and development history, therefore, our results are not easily generalized. Despite these limitations, the existence of a strong, self-sustaining and genetically diverse feral honey bee population in the city represents a genetic pool that can be important for beekeepers for future managed stocks and successful urban wildlife management and conservation in the cities. Urban areas offer more opportunities for conservation than was previously thought, and the research on urban insect pollinators is changing our concept of the biological value and ecological significance of urban areas, while, equally important, it provides us with the opportunity to estimate the importance of free-living *A. mellifera* in the urban ecosystem. Our study of this ecologically and agriculturally important species underscores the need for more data on the effects of urbanization on feral *A. mellifera* and potential changes in its ecology and evolution in urban habitats, on both local and global scales.

## Materials and methods

### Sampling

The sampling was carried out on the territory of the city of Belgrade (44° 49′ 14″ N and 20° 27′ 44″ E) (Fig. 6, Suppl. Fig. S8 and Suppl. Table S1). Belgrade is the capital and the largest urban area in the Republic of Serbia, with nearly 1.7 million permanent residents, approximately a quarter of all citizens of Serbia^65^. The territory of the city of Belgrade is 3.22268 km^2^ and the urban part covers around 1035 km^2^. The central part of the city covers 35996 km^2^^65^.

For the purpose of this study, we have collected samples from two types of honey bee colonies: feral and managed colonies (beehives from apiaries) with the participation of the Belgrade Beekeepers Society which collects the locations and citizen reports of requests for removal of honey bee colonies^32^. In the period 2020–2021, we have sampled 40 FCs and 7 different apiaries with 42 beehives (MCs). Sampled FCs represent the colonies that were able to survive independently for at least two years at the same location without the help of the beekeepers. From each sampled colony, we collected 5–8 worker bees that were immediately frozen and stored at − 20 °C. For genetic analysis, a worker bee was chosen as a representative sample for the colony.

For the comparison with other localities in Serbia, we used data from 241 samples from 46 apiaries from the countryside published in^40^ and^57^.

### DNA extraction and PCR–RFLP analysis

Whole-genomic DNA was extracted using the protocol described in^66^. PCR–RFLP method described in^61^ was used to distinguish mitochondrial lineages characteristic for *A. m. carnica* and *A. m. macedonica*. PCR amplification of the COI fragment and following digestion with *Nco*I and *Sty*I restriction enzymes was performed according to the protocol described in^40^.

### Microsatellite and fragment analysis

For comparison of different honey bee colonies at the autosomal level, we used the 14 most frequent microsatellite loci previously published in various studies. A detailed primer list and primer specification, together with a comprehensive protocol description, can be found in^57^.

### *tRNA*^*leu*^*-cox2* intergenic region sequencing

To determine the maternal relationship between different colonies, we have amplified and sequenced a segment of mitochondrial DNA known as the intergenic region located between the *tRNA*^*leu*^ and *cox2* genes^67^. The PCR program, primers used for the amplification of this region, and the sequencing protocol are described in^40^.

### Statistical analyses

All sequences used in the analyses were aligned using MEGA 10.0.4 software^68^. Standard parameters of genetic diversity for the intergenic region mtDNA *tRNA*^*leu*^*-cox2* and microsatellite loci (number of haplotypes, number of polymorphic sites, haplotype diversity, nucleotide diversity (π), number of alleles, number of alleles based on a minimal sample size (obtained by rarefaction), number of private alleles based on a minimal sample size (obtained by rarefaction), observed heterozygosity (H_O_) and expected (H_E_), random match probability (RMP) and the mean number of pairwise differences (MPD)) were calculated using the Arlequin ver. 3.5.2.2 software^69^ and HP-Rare 1.1^70^. The RMP parameter is used for expressing the probability that two randomly sampled individuals from a population have a matching genotype and is calculated as the sum of square frequencies^71^. The MPD is a parameter that represents the measure of differences between all pairs of haplotypes in the sample. The Arlequin ver. 3.5.2.2 software was used to assess genetic differentiation among populations by molecular variances analysis (AMOVA) and to estimate pairwise population and overall *F*_*ST*_ and *F*_*IS*_ values. The statistical significance of all tests performed was assessed with 10,000 permutations. The matrix of pairwise population *F*_*ST*_ values was visualized using the multidimensional scaling method (nonmetric MDS) implemented in the PAST 3.25 software^72^ and R functions connected with the Arlequin ver. 3.5.2.2 software. Hardy–Weinberg equilibrium (HW) was tested using Arlequin software with 1000,000 steps in MC and 100,000 dememorization steps. The effect of the recent bottleneck was also evaluated based on the Garza–Williamson index (G–W) which represents the ratio between the number of alleles and the allelic range and was calculated using the Arlequin software. Linkage disequilibrium between the pairs of loci was estimated using the likelihood ratio test in Arlequin with 10,000 numbers of steps in MCs and 10,000 dememorization steps.

### Phylogeography

Phylogeographic analysis was performed using 82 mtDNA haplotypes found in two different types of colonies of *A. mellifera* (Suppl. Table S1). The phylogeographic network was constructed using the median-joining method and maximum parsimony calculations for post-processing available in the software Network 10.2.0.0 (https://www.fluxus-engineering.com/network_terms.htm). Substitutions were equally weighted as no data of different evolutionary rates are available for the *tRNA*^*leu*^*-cox2* intergenic region for *A. mellifera*.

Substitutions specific for each haplotype were determined as the difference in nucleotide positions compared to the reference mitogenome NC_001566^73^.

### Population structure

The structure of the population was assessed using DAPC and STRUCTURE analysis. The number of genetic clusters represented in the FCs and MCs from Belgrade was estimated with STRUCTURE v 2.3.4 software^74–77^. For the analysis, the admixture model was used with a burn length of 10,000 and a Markov Chain Monte Carlo (MCMC) of 100,000 randomizations. The range of the possible number of clusters (K) ranged from 1 to 14, with a series of 10 runs for each K. The results obtained by STRUCTURE were analysed by the STRUCTURE harvester^78^. To detect the number of K groups that best fits the data set, this software uses results generated by the STRUCTURE software to create a plot of the mean likelihood value per K value and calculates the highest value of the second-order rate of change (Delta K) using the Evanno’s method^79^. The model choice criterion, LnP (D), implemented in the STRUCTURE which detects the true K as an estimate of the posterior probability of the data for a given K was also evaluated. The most likely scenario was chosen and used to graphically plot both the analysed individuals and populations.

A STRUCTURE-like approach assumes that markers are not linked and that populations are panmictic^77^, DAPC is a more convenient approach for populations that are clonal or partially clonal, and we used DAPC for the presentation of observed distances among samples. The DAPC method performs linear discriminant analysis (LDA) on the transformed matrix of principal component analysis (PCA)^80^. LDA was performed on the first 62 PCs, which cumulatively conserved 98.9% of the total variance. The number of PCs retained was estimated using randomly repeated cross-validation (100 iterations). Additionally, the PCA transformed matrix (all 197 PCs) was used to find the optimal number of clusters using Ward's method^81^.

### Coancestry

In order to assess the general gene flow among different colonies, we have estimated the relatedness, *r*, among the pair of colonies (dyads). Estimation was performed using the COANCESTRY program^41^. For this analysis, we used all seven relatedness estimators (TrioMl^42^; Wang^82^; LynchLi^83,84^; LynchRd^85^; Ritland^86^; QuellerGt^87^; DyadMl^88^) including three inbreeding estimators (TrioMl^42^; Ritland^86^; DyadMl^88^) implemented in the COANCESTRY^41^. Bootstrapping of 10,000 samples over loci was performed to obtain the 95% confidence interval (95CI) of each estimator for each dyad. The inbreeding coefficient (*F*) for each honey bee colony was estimated using the same parameter settings. We used the following threshold values of the *r*_*xy*_ coefficient: *r*_*xy*_ values lower than 0.09375 marked unrelated colonies, *r*_*xy*_ values lower than 0.1875 and higher or equal to 0.09375 were considered as third-order relatives (relationship), *r*_*xy*_ values lower than 0.375 and higher or equal to 0.1875 were considered as second-order relatives, *r*_*xy*_ values higher or equal to 0.375 were considered as having full-sibling or parent–offspring relationship^85,89^. ML-Relate^70^ software package was used to confirm acquired parent-offspring, full-siblings, and half-sibling relationships.

## Supplementary Information

Supplementary Figures.

Supplementary Tables.

## Data Availability

The authors declare that the data supporting the findings of this study are available in the article and its supplementary information files. Source data are provided in this paper. The datasets generated during the current study are available in the GenBank repository, https://www.ncbi.nlm.nih.gov/genbank/ [accession numbers: ON187787-ON187868].
