# Supplementary material for: Urban ecosystem drives genetic diversity in feral honey bee
Source: Sci Rep. 2022 Oct 21;12:17692. doi: 10.1038/s41598-022-21413-y (PMC9587283; doi:10.1038/s41598-022-21413-y)
Supplement: Supplementary file 1 — Supplementary Figures. [file 41598_2022_21413_MOESM1_ESM.docx]

**Urban ecosystem drives genetic diversity in feral honey bee**

**Aleksandra Patenković^1^*, Marija Tanasković^1^, Pavle Erić^1^, Katarina Erić^1^, Milica Mihajlović^2^, Ljubiša Stanisavljević^3^ and Slobodan Davidović^1^**

^1^ Department of Genetics of Populations and Ecogenotoxicology, Institute for Biological Research “Siniša Stanković” – National Institute of the Republic of Serbia, University of Belgrade, Bulevar despota Stefana 142, 11060 Belgrade, Serbia

^2^ Center for Forensic and Applied Molecular Genetics, Faculty of Biology, University of Belgrade, Studentski trg 16, 11000 Belgrade, Serbia

^3^ Center for Bee Research, Faculty of Biology, University of Belgrade, Studentski trg 16, 11000 Belgrade, Serbia

* aleksandra@ibiss.bg.ac.rs

**ABSTRACT**

Urbanization can change biodiversity in both directions, positive and negative, and despite the rising global trend of urban beekeeping, little is known about the impact of urbanization on the genetic diversity of honey bees. We investigate how urbanization affects the genetic variability of feral and managed honey bee colonies that are spread throughout the entire city, even in highly urban areas, through genetic analysis of 82 worker bees. We found convincing evidence of high genetic differentiation between these two groups. Additionally, by comparing city samples with 241 samples from 46 apiaries in rural parts of the country, variations in COII mtDNA, tRNAleu-cox2, and microsatellite loci indicated that feral colonies have distinct patterns of genetic diversity. These results, with evidence that feral honey bees find niches within highly modified and human-dominated urban landscapes, lead us to conclude that urbanization is a driver of the genetic diversity of feral honey bees in the city.

**Funding**

This research was funded by the Science Fund of the Republic of Serbia, PROMIS, Grant No. 6066205, SERBHIWE for A.P., M.T., P.E., K.E., Lj.S., S.D. and the Ministry of Education, Science and Technological Development of the Republic of Serbia, Grant number 451-03-68/2022-14/ 200007 for M.T., P.E. A.P., K.E. and S.D.

**Contributions**

S.D., A.P. Lj.S and M.T. designed the study. A.P., M.T., P.E., K.E. and S.D. collected the original field data; P.E., K.E., A.P., M.T., M.M. and S.D. performed analyses; M.T., P.E. and S.D conducted the bioinformatics and statistical analyses; A.P. wrote the manuscript with comments and suggestions from S.D. All authors have read, revised, and approved the manuscript for publication. S.D. and Lj.S. jointly supervised this work.

(a)

**
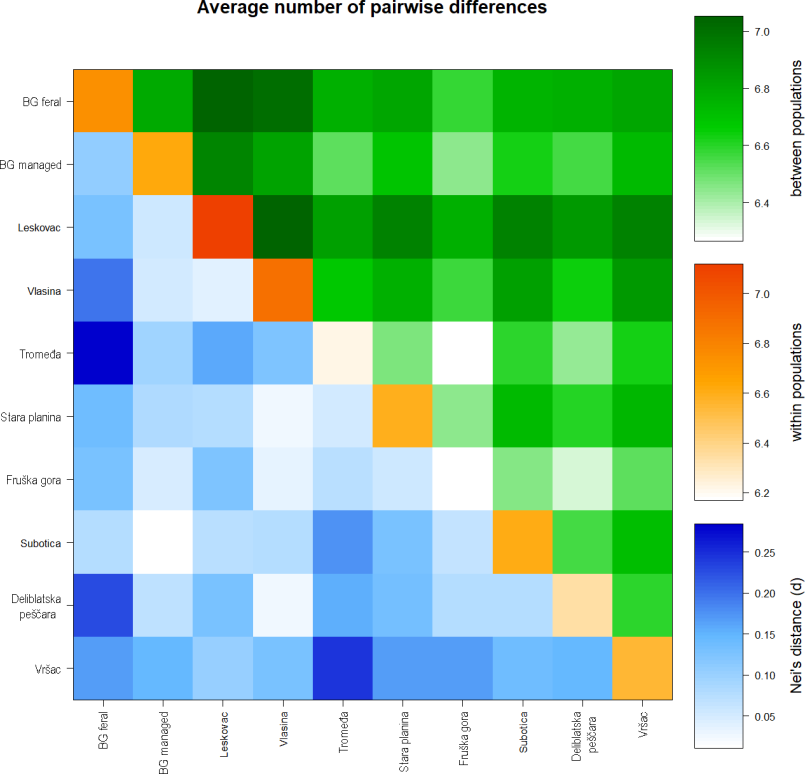
**

(b)

**
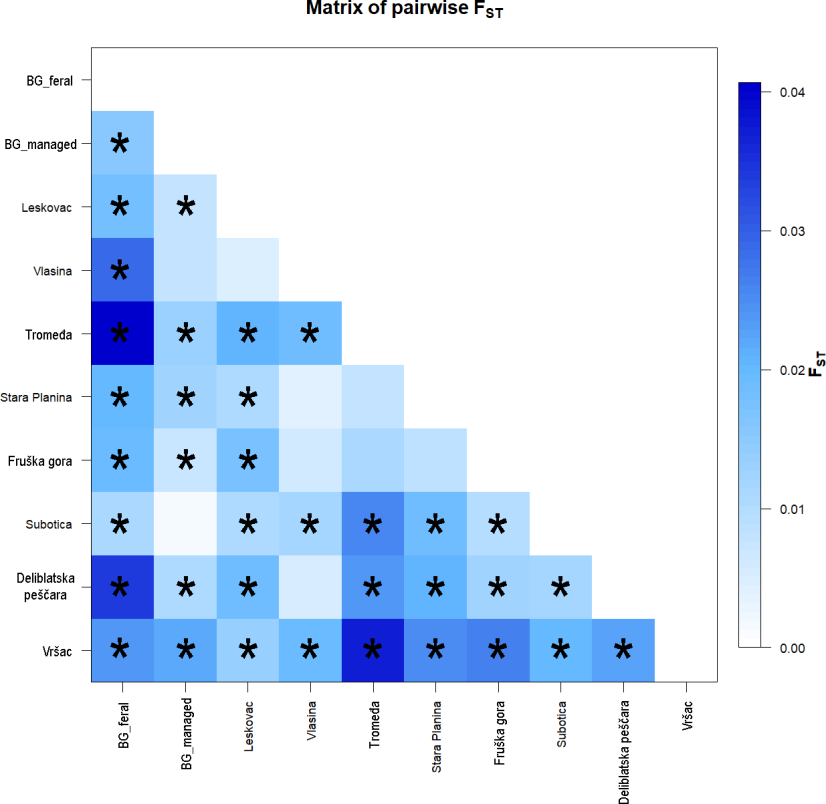
**

**Suppl. Figure S1.** Matrices of the average number of pairwise, Nei's (a) and *F_ST_* (b) distances based on the analysis of 14 microsatellite loci for two types of honey bee colonies from Belgrade (feral and managed) and colonies from other localities in Serbia. a) The average number of pairwise differences between populations is presented above diagonal, the average number of pairwise differences within the population is presented diagonal and Nei's distances are presented below diagonal. b) Statistically significant *F_ST_* values are marked with an asterisk (*).

**
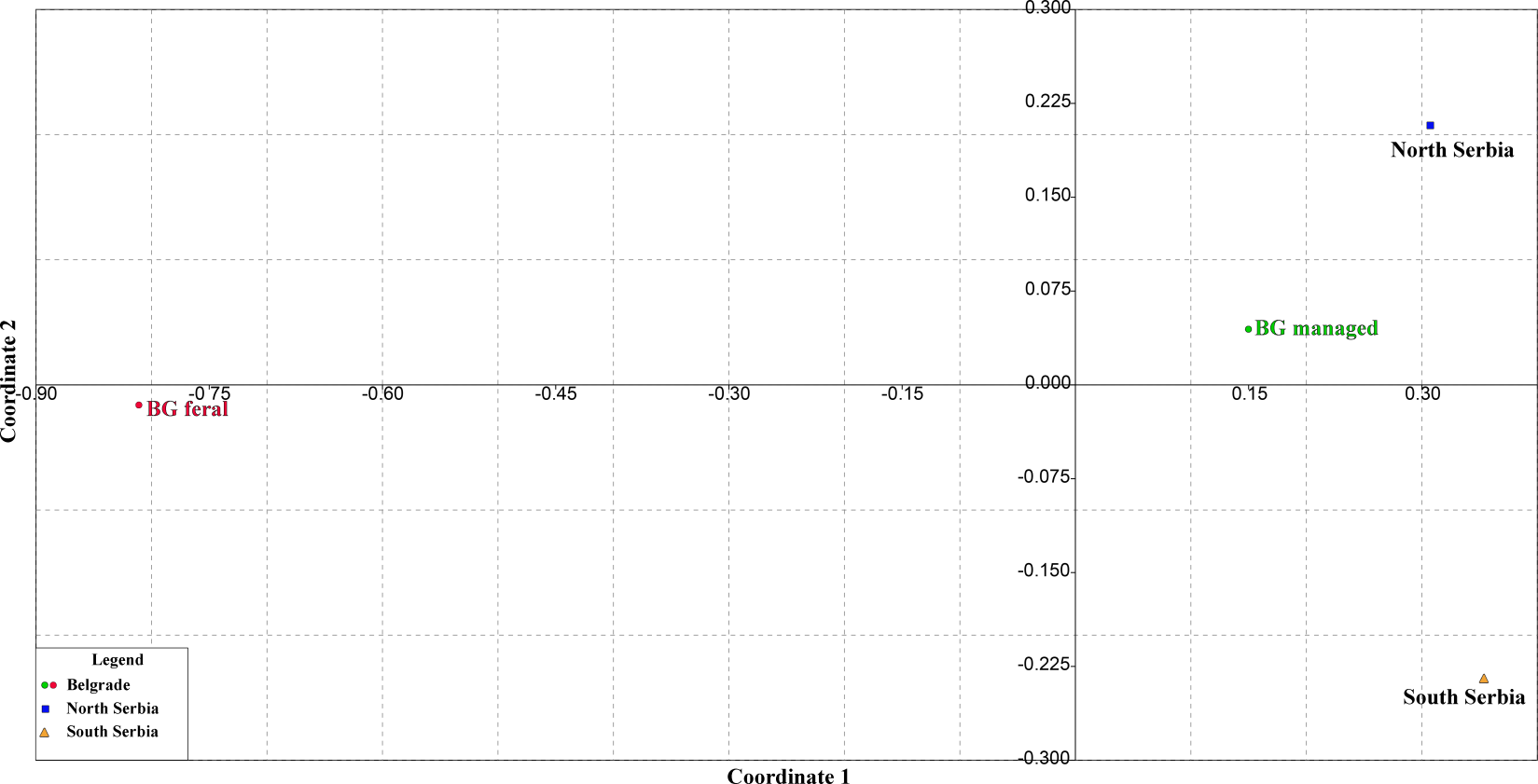
**

**Suppl. Figure S2.** Non-metric multidimensional scaling plot of *F_ST_* distances between two types of honey bee colonies (feral and managed) from Belgrade and honey bee colonies originating from North and South parts Serbia based on the variability of 14 microsatellite loci. The goodness of fit is expressed with the stress value, which is 0.0000 for this data set. Population pairwise *F_ST_* values are presented in Suppl. Table S7.


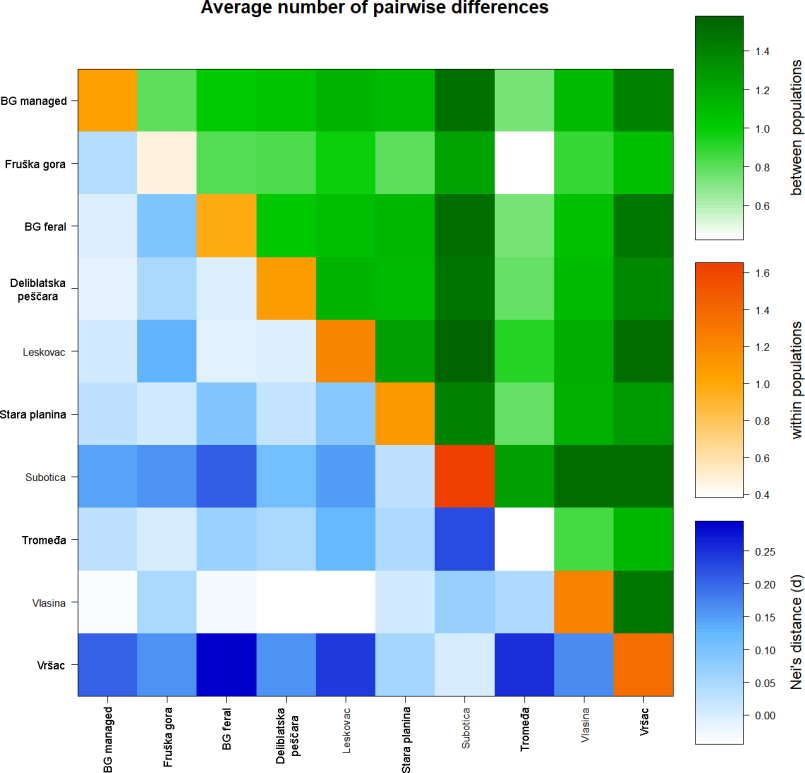

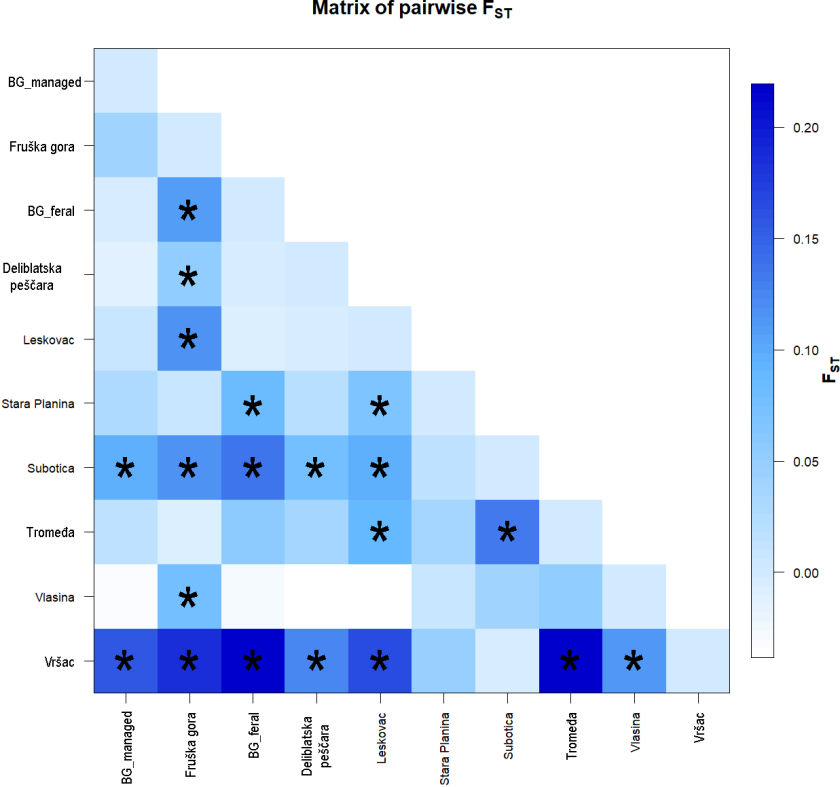


(a) (b)

**Suppl. Figure S3.** Matrices of the average number of pairwise, Nei's (a) and *F_ST_* (b) distances based on the analysis of *tRNA^leu^-cox2* intergenic sequence variability for two types of honey bee colonies from Belgrade (feral and managed) and colonies from other localities in Serbia. a) The average number of pairwise differences between populations is presented above diagonal, the average number of pairwise differences within the population is presented diagonal and Nei's distances are presented below diagonal. b) Statistically significant *F_ST_* values are marked with an asterisk (*).

**
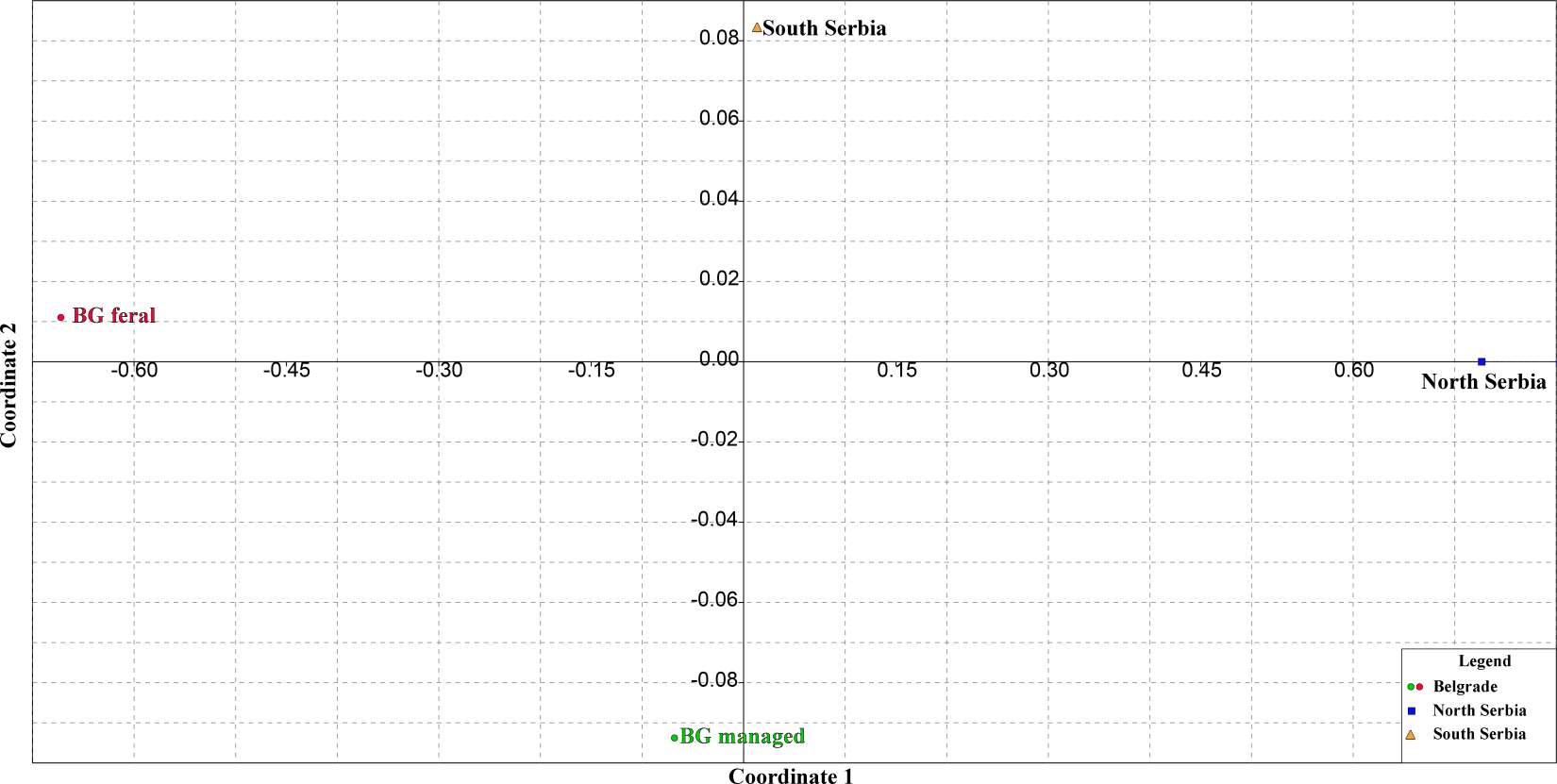
**

**Suppl. Figure S4.** Non-metric multidimensional scaling plot of *F_ST_* distances between two types of honey bee colonies (feral and managed) from Belgrade and honey bee colonies originating from North and South parts Serbia based on the variability mtDNA *tRNA^leu^-cox2* intergenic region sequence. The goodness of fit is expressed with the stress value, which is 0.0000 for this data set. Population pairwise *F_ST_* values are presented in Suppl. Table S8.

Link to the 3D figure:

<https://radar.ibiss.bg.ac.rs/bitstream/handle/123456789/4954/Figure%20S5.html?sequence=1&isAllowed=y>

**Suppl. Figure S5.** Discriminant analysis of principal components in which LDA was performed on the first 62 PCs (out of 197 PCs) which cumulatively conserve 99.8% of the total variance. The first three linear discriminants are presented in the plot: feral and managed colonies from Belgrade with different localities from South (Leskovac, Vlasina, Stara planina) and North (Fruška gora, Deliblatska peščara, Subotica, Vršac) Serbia.

This 3D image is deposited in the RADAR Repository, and more information about the image itself is available at this link: <https://radar.ibiss.bg.ac.rs/handle/123456789/4954>


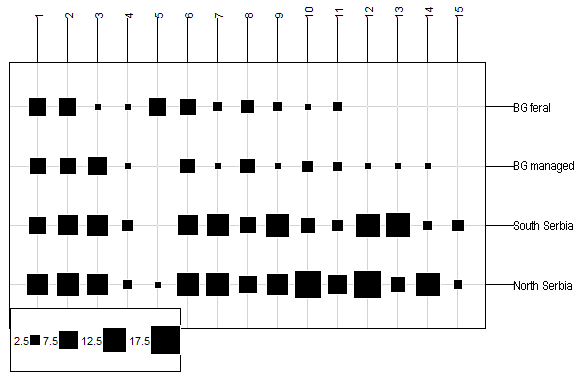


**Suppl. Figure S6.** Distribution of clusters according to the DAPC method and inferred number of 15 clusters.


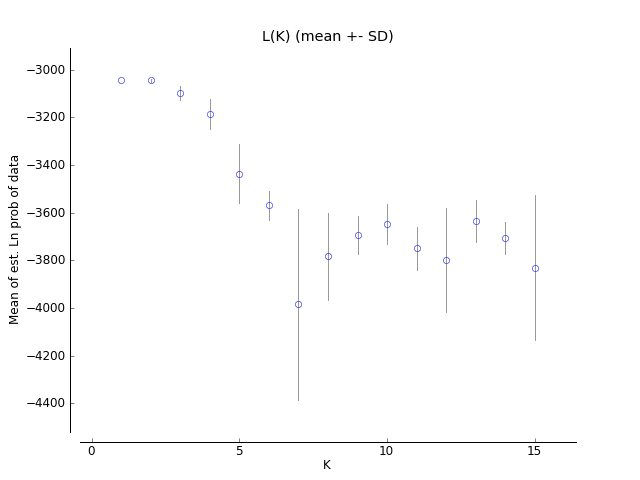


**Suppl. Figure S7.** L(*K*) mean for the assumed number of genetic clusters.

**
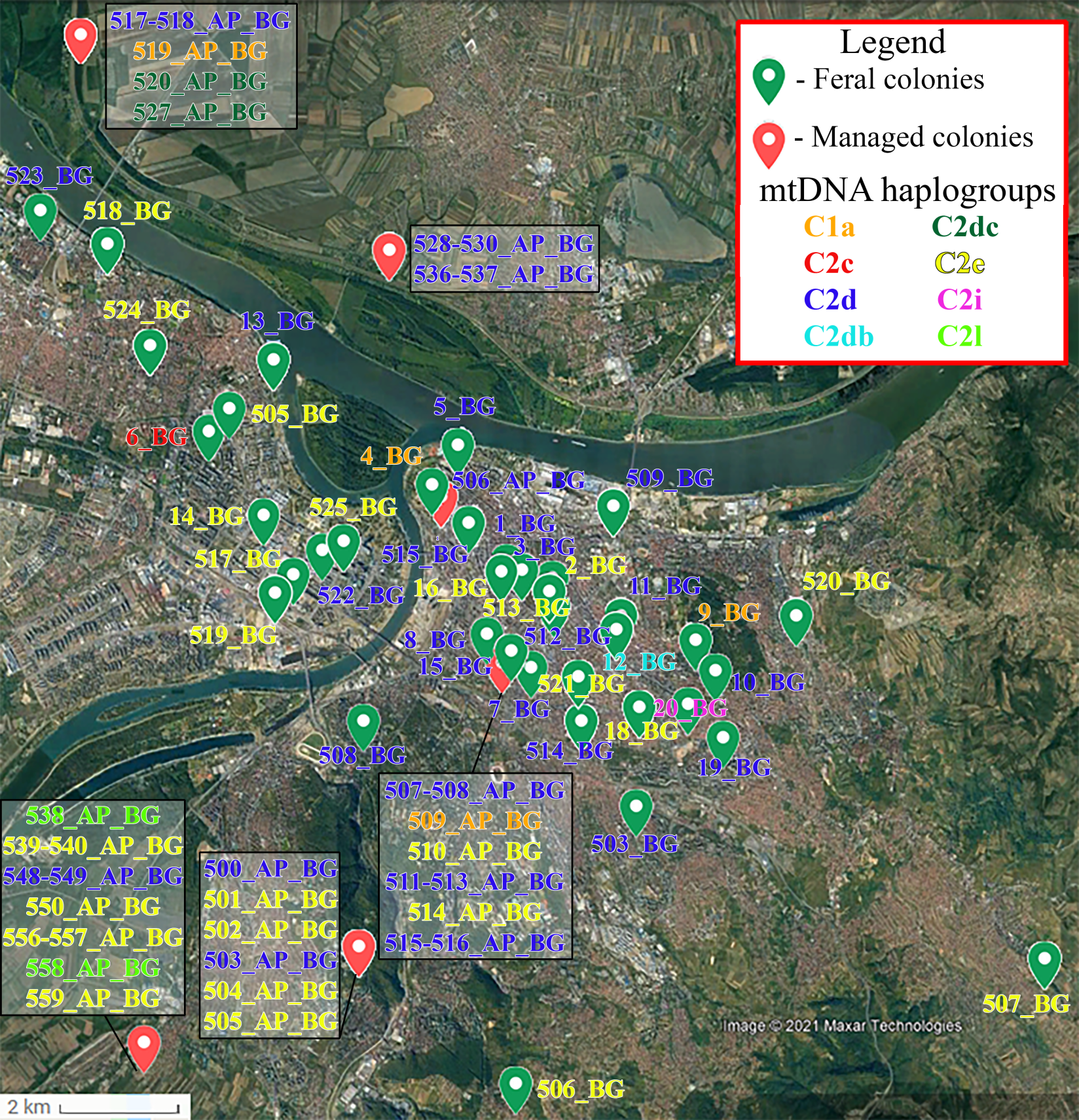
**

**Suppl. Figure S8.** Sampling locations for feral and managed honey bee colonies in Belgrade with the detected mtDNA haplotypes. The base satellite imagery was obtained from Google Earth, and Adobe Photoshop CC 2015 (https://www.adobe.com/products/photoshop.html) was used to map the data by S. D. The locations overlying the map were obtained from the field using a GPS device (model no: Garmin eTrex 22x).
